# Protocol for microinjection of rapamycin into the zebrafish habenula

**DOI:** 10.1016/j.xpro.2024.103566

**Published:** 2025-01-11

**Authors:** Olga Doszyn, Tomasz Dulski, Justyna Zmorzynska

**Affiliations:** 1 Laboratory of Developmental Neurobiology, International Institute of Molecular Mechanisms and Machines, 02-247 Warsaw, Poland; 2 Laboratory of Molecular and Cellular Neurobiology, International Institute of Molecular and Cell Biology in Warsaw, 02-109 Warsaw, Poland

**Keywords:** Developmental biology, Model Organisms, Neuroscience

## Abstract

Mechanistic target of rapamycin complex 1 (mTorC1) activity plays a crucial role in brain development. Here, we present an approach for rapamycin microinjection into the habenula of larval zebrafish to achieve localized inhibition of the mTorC1 pathway and explore the role of mTorC1 in habenula function. We describe steps for performing microinjections and maintaining zebrafish larvae before and after the procedure.

For complete details on the use and execution of this protocol, please refer to Doszyn et al.^1^

## Highlights

• Instructions for husbandry of zebrafish larvae
• A step-by-step guide for microinjection of drugs into the zebrafish habenula
• Evaluation of rapamycin microinjection efficacy
• Localization of the mTorC1 pathway inhibition in the brain

## Abstract (editor highlights)

Publisher’s note: Undertaking any experimental protocol requires adherence to local institutional guidelines for laboratory safety and ethics.

## Graphical abstract

**Figure:**
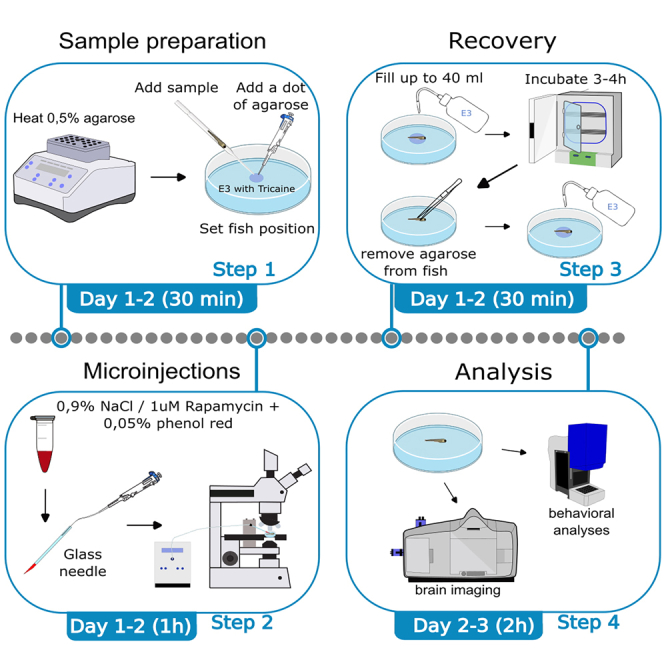

## Before you begin

Mechanistic target of rapamycin complex 1 (mTorC1), integrates multiple extra- and intracellular signals in order to regulate transcription, translation, protein degradation and cytoskeleton dynamics. In the brain, it controls proliferation, migration, differentiation, synaptogenesis, and neuronal activity.^2^ This protocol describes the procedure for injecting rapamycin, the direct mTorC1 inhibitor, into the zebrafish habenula, a bilateral structure located in the dorsal diencephalon. The morphology and function of the habenula is asymmetric, with the left and right habenula playing different roles in the integration of various sensory stimuli and regulation of behavioral response.^3^^,^^4^ This protocol allows for localized mTorC1 inhibition. We have examined the role of mTorC1 in the light-preference behavior of *tsc2*^*vu242/+*^ zebrafish, which is regulated by the left habenula.^4^ However, depending on the researcher’s subject of interest, this protocol can also be adapted for microinjections of different drugs, as well as for other zebrafish lines. In such cases, it might be necessary to adjust parameters such as drug concentration or age of injected larvae; the technical aspects of the injection itself would remain the same.

### Institutional permissions

For the procedure described here, the *tsc2*^*vu242/+*^ zebrafish line was used. All experiments performed were conducted in accordance with the Act of 15 January 2015 on the protection of animals used for scientific and educational purposes, Directive 2010/63/EU of the European Parliament and of the Council of 22 September 2010 on the protection of animals used for scientific purposes and were approved by the Animal Welfare Commission of the IMol and the IIMCB.

As this protocol uses zebrafish younger than 5 days post-fertilization (dpf), it does not require the additional permission of local ethical committee.

### Preparation of buffers and stock solutions

**Timing: 1 h**

1. Prepare the 60× E3 medium stock solution (recipe to be found in materials and equipment). Autoclave and keep at 4°C.
  a. Prepare the E3 medium by diluting the stock to 1× in MilliQ H_2_O.
2. Prepare the Tricaine stock solution (recipe to be found in materials and equipment). Keep at 4°C in the dark.
3. Prepare a 1% solution of low melting point agarose in E3 medium.

***Note:*** If the solution is being prepared prior to the day of the experiment, it can be stored at 4°C and melted as needed. If prepared immediately before injections, keep in liquid form at 55°C–60°C.

4. Prepare a 0.9% solution of NaCl. Store in room temperature for up to a week or in 4°C for a longer time. Bring to room temperature or keep in the incubator with the fish at 28.5°C before use.
5. Prepare a 1 μM rapamycin solution in 0.9% NaCl. Aliquot and keep at −20°C. Bring to room temperature or 28.5°C before use.

### Zebrafish breeding

**Timing: 1–2 h per day for 4 consecutive days**

6. Set up a spawning tank with male and female fish (either separated by a divider to be removed on the next-day’s morning, or in a light-controlled room where the light will be off during the night and will switch on in the morning).
7. On the following day, collect the fertilized eggs. Place up to 60 eggs per a Ø100 mm Petri dish filled with 40 mL of E3 medium. Incubate at 28.5°C, with a diurnal cycle of 14 h light/10 h darkness.
8. On the first and second day post fertilization (dpf), remove any debris, dead embryos or unfertilized eggs, and exchange the E3 medium.

### Preparation of micropipettes

**Timing: 30 min**

9. Prepare the micropipettes.
  a. Insert a glass capillary into the heat puller and prepare the needles as described by Konadu et al.^5^
  b. Take a 10-cm-long borosilicate glass capillary (1.0 mm outer diameter, thin-walled) placed in the micropipette puller (e.g., from Sutter Instrument Company).
  c. Carefully load the capillary tube into the carriage, aligning it precisely within the groove and securing it with clamps.
  d. Glide the capillary through the heating filament to ensure consistent alignment across the entire length.
  e. Once positioned, initiate the pull program with settings tailored to heat, pull, velocity, delay, and pressure.
    ***Note:*** After the pull process, the needle tip is formed at both ends that has to be broken open using fine forceps under a stereo microscope.
  f. Using fine forceps under a stereo microscope with good optics (at least 150× magnification), snap off the pointed end to obtain a micropipette with an open tip of ∼3 μm.
    **CRITICAL:** Use microscope calibration slide with ruler of 0.01 mm or microscope reticle to measure tip diameter.
  g. Load the micropipette with 3 μL of 0.9% NaCl or rapamycin solution.
  h. Insert the micropipette into the injector pump holder.
  i. Put a drop of mineral oil on the calibration slide.
  j. Using the calibration slide with a ruler of 0.01 mm set the drop size to 0.5 nL by injecting a drop to the mineral oil, measuring it and adjusting the size with pressure.

**Figure 1:**
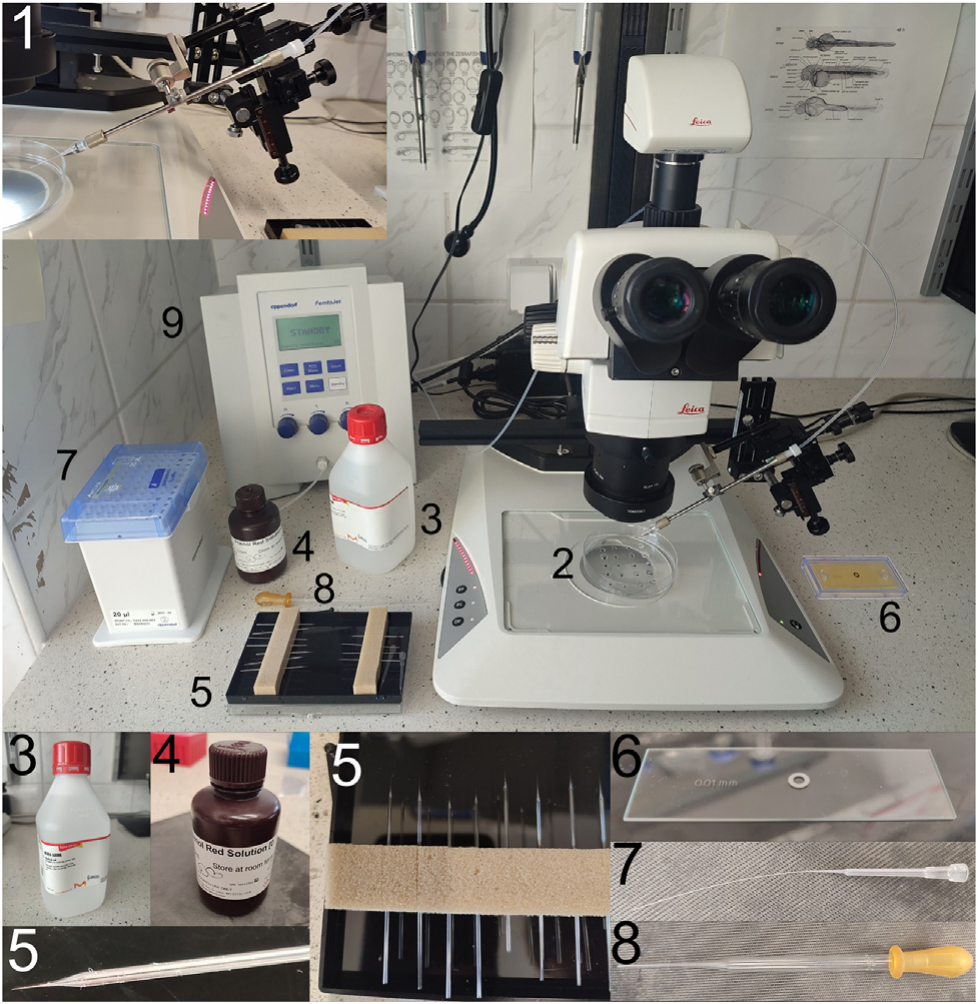
Workstation for injection 1. Glass needle mounted on the injector pump holder. 2. Petri dish with fish mixed in each dot with E3 and agarose. 3. Mineral oil necessary for injection calibration. 4. Phenol Red solution. 5. Glass needles. 6. Microscope calibration slide. 7. Extended pipette tips for easy needle loading. 8. Pasteur pipette. 9. Injection pump.

Workstation for microinjection with the necessary equipment is presented on Figure 1. Troubleshooting 1.

## Key resources table

**Table:** 

REAGENT or RESOURCE	SOURCE	IDENTIFIER
**Chemicals, peptides, and recombinant proteins**		

NaCl (sodium chloride)	Chempur	Cat# 117941206
KCl (potassium chloride)	Chempur	Cat# 117397402
CaCl_2_ (calcium chloride)	Chempur	Cat# 118748703
MgCl_2_ x 6H_2_O (magnesium chloride hexahydrate)	Chempur	Cat# 116120500
Tricaine	Sigma-Aldrich/Merck	Cat# E10521
Tris base	Carl Roth	Cat# 4855.3
HCl	Chempur	Cat# 115752837
Rapamycin	Sigma-Aldrich/Merck	Cat# 553210
Phenol red	Sigma-Aldrich/Merck	Cat# P0290
TopVision low melting point agarose	Thermo Fisher Scientific	Cat# R0801

**Experimental models: Organisms/strains**		

Zebrafish: tsc2^vu242/+^ (mixed background) larvae; sex not specified yet	Kim et al.^6^	RRID:ZFIN_ZDB-GENO-180906-3

**Other**		

Dry block thermostat Bio TDB-100	Biosan	Cat# BS-010412-AAA
Borosilicate glass capillaries (outer diameter 1.0 mm / inner diameter 0.75 mm)	Sutter Instrument	Cat# BF150-75-10
Flaming/Brown micropipette puller	Sutter Instrument	Cat# P-1000
Microscope Leica M165 FC 198× magnification	Leica Microsysytems	https://www.leica-microsystems.com/products/light-microscopes/stereo-microscopes/p/leica-m165-fc/
Injector pump FemtoJet 4i	Eppendorf	Cat# E5252000021

## Materials and equipment

**Table:** 60× E3 medium stock solution

Reagent	Final concentration	Amount
NaCl	297 mM	17.4 g
KCl	10.7 mM	0.8 g
CaCl_2_	19.6 mM	2.18 g
MgCl_2_ x 6H_2_O	24 mM	4.89 g
MilliQ H_2_O	N/A	fill up to 1 L
**Total**	**N/A**	**1 L**

Can be stored long term at 4°C.

**Table:** Tricaine stock solution

Reagent	Final concentration	Amount
Tricaine	15.3 mM	1 g
1M Tris-HCl pH 9	21 mM	5.25 mL
MilliQ H_2_O	N/A	244.75 mL
**Total**	**N/A**	**250 mL**

Can be stored long term at 4°C in the dark.

## Step-by-step method details

### First microinjection

**Timing: 2 h**

This step describes the preparation of zebrafish larvae and the microinjection procedure. For rapamycin injections into the left habenula, it was performed at 3 dpf and then repeated again at 4 dpf, as this was previously established to be required for the sustained effect of the treatment. Troubleshooting 2.

1. Heat the 1% agarose solution to melt. Keep it in the heat block at 55°C–60°C.
2. Prepare the 0.9% NaCl and 1 μM rapamycin solutions. Add 1 μL of 0.05% Phenol Red per every 10 μL of solution for visualization.
3. Add Tricaine solution into the E3 medium with the larvae to a final concentration of 0.01%.

***Note:*** It is recommended to sedate a smaller number of larvae at a time, perform the microinjection procedure to the end, and repeat for the next batch.

4. Place single larva in one drop of approximately 20 μL of E3 with Tricaine on a Petri dish.
  ***Note:*** You can put as many separate drops as you manage to inject (e.g. 20 per dish). Pipette an equal volume (approx. 20 μL) drop of 1% agarose solution (to the final concentration of 0.5%) onto the drop with the larva and position the larva with the dorsal side of the head facing up.
  a. Place the dish under the microscope. If necessary, using a thin pipette tip, carefully manipulate the larva to adjust its position, ensuring the habenulae are easily accessible.
  b. After the agarose solidifies, pipette a drop of E3 medium with Tricaine on top, ensuring that the fish is continuously sedated and submerged in water.
    **CRITICAL:** To increase efficiency of the procedure, several larvae can be mounted on a single dish, and injected in a batch. Extra attention must be then paid to ensure the E3 medium covering the larvae does not evaporate before the end of the injections. Add drops of fresh E3 medium with Tricaine when needed.
5. Insert the micropipette tip between the habenula and the surrounding blood vessel.

**Figure 2:**
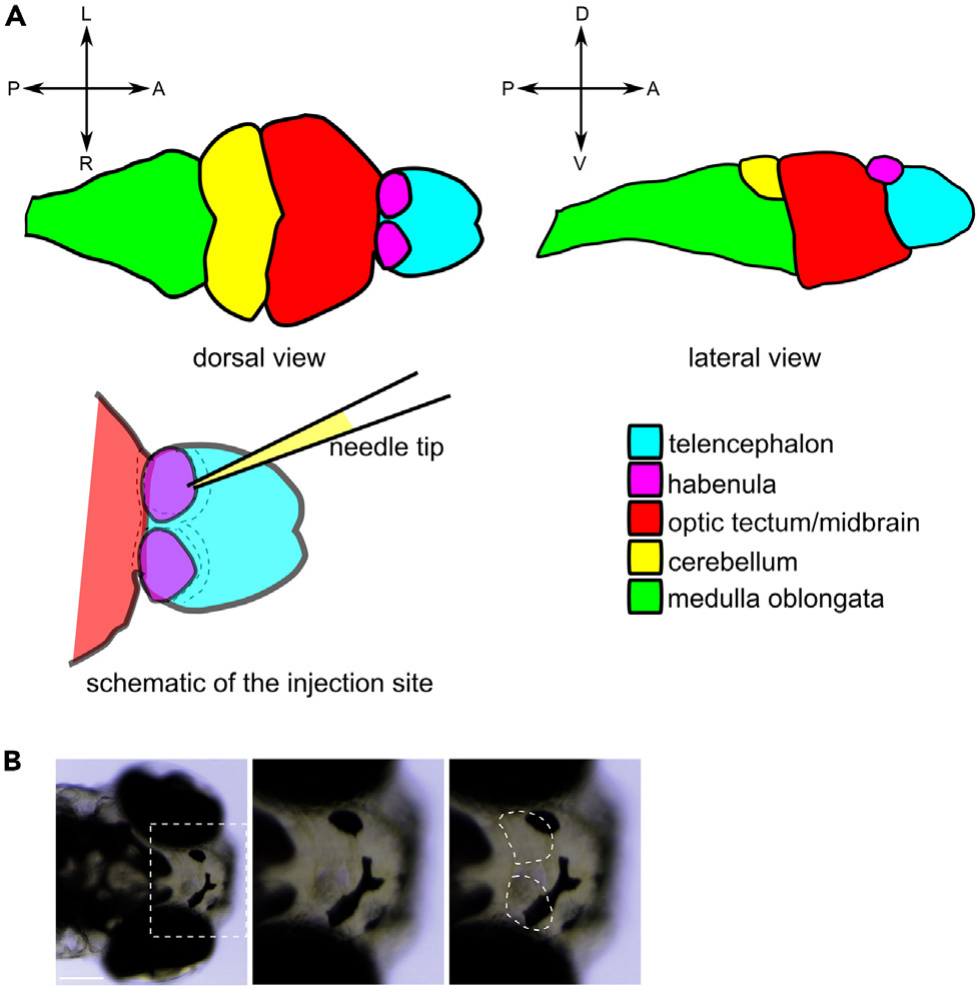
Habenula localization within the zebrafish brain (A) Schematic of the zebrafish habenulae location within the brain depicting also the injection site. Surrounding blood vessels are marked with dashed lines. A – anterior, P – posterior, L – left, R – right, D – dorsal, V – ventral. (B) An exemplary images of the zebrafish head with zoom-in onto the habenulae. Dark spots are melanophores in the skin. Scale bar – 200 μm.

***Note:*** Blood vessels should be easily visible under stereo microscope because the blood cells are floating in them (Figure 2.)

6. Administer 4 drops of NaCl with Phenol Red or rapamycin with Phenol Red solutions by injecting 1 drop at a time at 20 s intervals. Troubleshooting 3.

***Note:*** For microinjection use the stereo microscope with at least 150× magnification.

### Recovery

**Timing: 3–4 h + 1 day**

This step describes the recovery procedure after the injections.

7. After each batch of injections, fill the Petri dish containing immobilized larvae with 40 mL of E3 (without Tricaine), and place it back in the incubator.
8. After 3–4 h, if the larvae did not freed themselves already, gently remove the agarose from around the larvae using a thin pipette tip. Troubleshooting 4.
  a. Transfer the free larvae to a clean Petri dish with fresh E3 medium.

***Note:*** Do not remove the agarose from the fish immediately after injection. Leaving the fish in agarose reduces the risk of damaging the fish. The fish can rest and recover after injection. Once they start moving, most of the fish can get out of the remaining agarose on their own. If not, be careful during removing the agarose with a tip, as the yolk sac can be easily damaged.

### Second microinjection and recovery

**Timing: 6 h + 1 day**

This step is required in the case of rapamycin injections, in order to achieve sustained effect of the treatment.

9. On the following day (4 dpf), perform the microinjections again by repeating steps 1-6.
10. Allow the larvae to recover as described in steps 7-8.
11. On 5 dpf, the larvae can be used for further experiments, such as behavioral testing, brain imaging, or immunofluorescence staining.

## Expected outcomes

**Figure 3:**
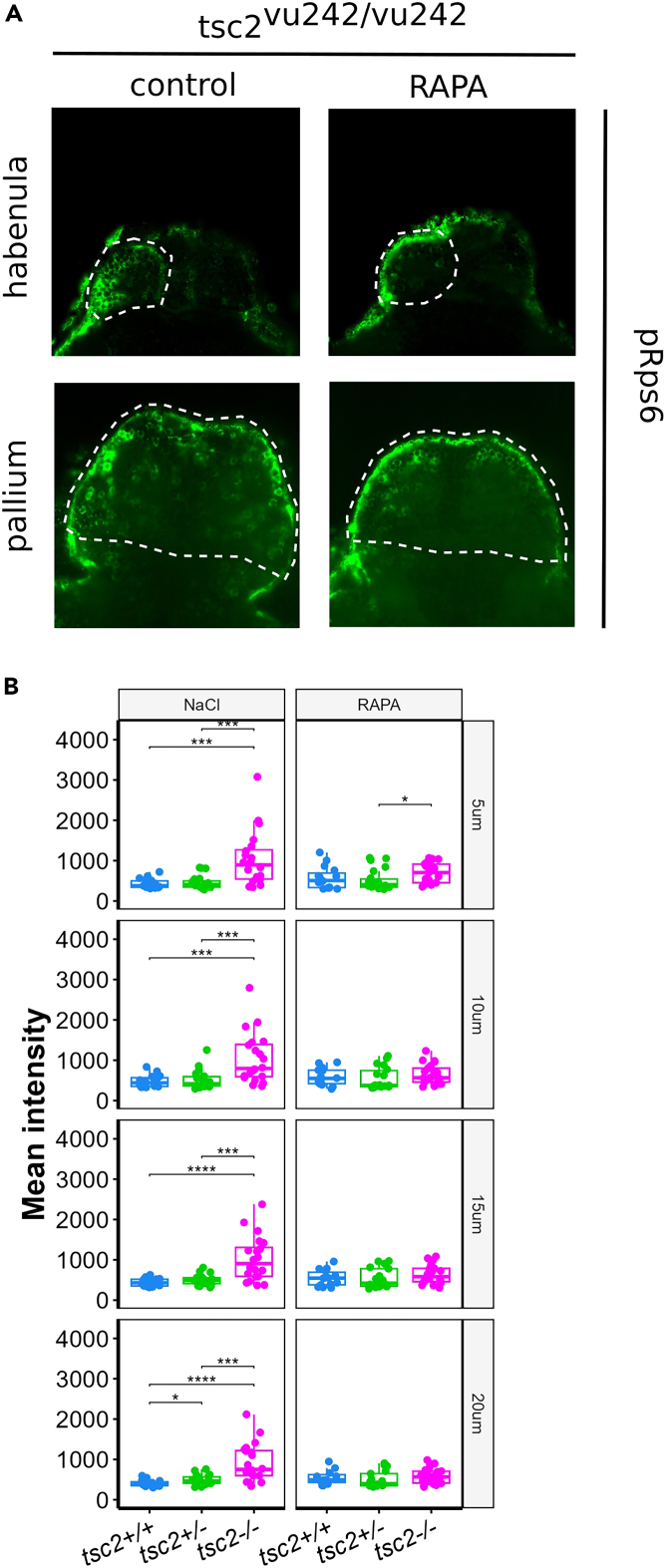
Expression levels of the pRps6 in the left habenula of tsc2^vu242/vu242^ zebrafish (A) Representative optical sections through habenula and pallium of *tsc2*^*vu242/vu242*^ fish injected at 3 and 4 dpf with NaCl or rapamycin, following immunofluorescence staining against pRps6. The images show a decrease in fluorescence intensity in the left habenula following rapamycin injections, but no significant change in fluorescence intensity in the pallium. (B) Quantification of mean intensity of pRps6 fluorescence from LdHb of *tsc2*^*vu242*^ fish injected at 3 and 4 dpf with NaCl or rapamycin (5 μm from the top of the habenula: *tsc2*^*vu242/vu242*^ control vs. *tsc2*^*+/+*^ control *p* = 0.000107 and vs. *tsc2*^*vu242/+*^ control *p* = 0.000244; *tsc2*^*vu242/vu242*^ treated with RAPA vs. *tsc2*^*vu242/+*^ treated with RAPA *p* = 0.022. 10 μm: *tsc2*^*vu242/vu242*^ control vs. *tsc2*^*+/+*^ control *p* = 0.000918 and vs. *tsc2*^*vu242/+*^ control *p* = 0.000867. 15 μm: *tsc2*^*vu242/vu242*^ control vs. *tsc2*^*+/+*^ control *p* = 8.73e-05 and vs. *tsc2*^*vu242/+*^ control *p* = 0.000456. 20 μm: *tsc2*^*vu242/vu242*^ control vs. *tsc2*^*+/+*^ control *p* = 7.5e-06 and vs. *tsc2*^*vu242/+*^ control *p* = 0.000676; *tsc2*^*vu242/+*^ control vs. *tsc2*^*+/+*^ control *p* = 0.047). The dots on the boxplots represent the number of fish in the experiment (*N* > 10 per experimental group).

The subsequent injections of 1 μM rapamycin at 3 and 4 dpf result in the inhibition of the mTorC1 activity localized to the injected region (left habenula). This can be verified by analyzing the phosphorylation levels of one of the target proteins of mTorC1, Rps6. Immunofluorescence staining shows lowered levels of phosphorylated Rps6 (pRps6) in the left habenula of *tsc2*^*vu242/vu242*^ mutants at 5 dpf following rapamycin injections, in comparison to control group injected with NaCl, bringing them down to a level comparable with that found in the wild-type fish (Figure 3). Additionally, we have previously shown that the *tsc2*^*vu242/vu242*^ mutants, compared to wild-type siblings, display a lowered preference for light over dark environment in the light-dark choice assay. This aberrant behavioral response to light was rescued by rapamycin injections into the left habenula.^1^

## Limitations

One limitation of this protocol is that it was optimized specifically to inhibit mTorC1 activity by rapamycin, thus, when using other drugs, the dose and timing of the administered drug should be tested prior to further experiments, especially as it might be different than when the drug is administered into the water. In our study, we have determined that two subsequent injections of 1 μM rapamycin at 3 and 4 dpf are required for effective inhibition of mTorC1 activity at 5 dpf, as a lower dose or a single injection did not result in significantly lowered levels of pRps6.^1^ Similarly, efficacy of the treatment should be tested when considering injections of other drugs, or at other developmental stages. This protocol might also be used for injections into other parts of the brain, e.g., the cerebellum. However, due to the lack of stereotaxic instruments designed for zebrafish larvae, finding a very specific region of interest might require additional effort and care. Additionally, while the habenulae are positioned dorsally and easily accessible, injecting into the deeper parts of the brain might be more problematic.

## Troubleshooting

### Problem 1

The ejected drops are too big or too small (related to preparation of micropipettes). The size of drops should be set to 0.5 nL. To set appropriate size use the microscope calibration slide with a ruler of 0.01 mm or microscope reticle.

### Potential solutions

• If the drop size is smaller than 0.5 nL, consider increasing the pressure in the injection pump. Do not increase the pressure too much (above 500 hPa), as you can damage the fish. If this solution will not resolve the problem, break the needle a little more under the stereoscope using the high magnification (at least 50×).
• If the drop size is bigger than 0.5 nL, consider decreasing the pressure in the injection pump. If this solution will not resolve the problem, then the diameter of the needle may be too big. Prepare another needle, breaking the tip off at a lower point. Always, break the needle under the stereoscope using the high magnification (at least 50× magnification).

### Problem 2

No effect of the drug is observed following the injection (related to expected outcomes).

### Potential solutions

• Consider increasing the dose of the drug.
• Multiple injections on subsequent days might be required. With multiple injections especially, take extra care to make sure they don’t cause sustained injury, and that the larvae have sufficient time to recover between injections.

### Problem 3

No drops are ejected from the needle (related to first microinjection).

### Potential solutions

• Check for air bubbles or any debris that might be blocking the needle.
• Check for any breaks or leaks in the needle or injector tubing.

### Problem 4

High mortality of larvae following injections (related to first microinjection and to recovery). The mortality rate depends on many factors like injection time and drug dose. However, the critical factor is the user. For unskilled users the mortality rate can reach up to 50% whereas for skilled users mortality rate decreases to ∼1%. Practice with wild-type larvae before your experiment to master the procedure.

### Potential solutions

• The most likely cause is damage to the body of the fish or rupturing the yolk during either mounting (step 4a) or freeing the fish from agarose (step 8). During those steps, take extra care not to damage the fish.
• The fish might also be damaged when the micropipette is inserted too deep into the tissue, or at the wrong place. Make sure you have located the habenula correctly, and that the fish is mounted in such a way that it is easily accessible, facing straight up.

## Resource availability

### Lead contact

Further information and requests for resources and reagents should be directed to and will be fulfilled by the lead contact, Justyna Zmorzynska (j.zmorzynska@imol.institute).

### Technical contact

Technical questions on executing this protocol should be directed to and will be answered by the technical contact, Justyna Zmorzynska (j.zmorzynska@imol.institute).

### Materials availability

This study did not generate new unique reagents. The fish mutant and transgenic lines are protected under material transfer agreement with the institutions that generated the lines. Upon appropriate agreement with these institutions, they can be requested from the lead contact.

### Data and code availability

This study did not generate datasets or codes.

## Acknowledgments

We thank Kevin Ess (Vanderbilt University) for the *tsc2*^*vu242/+*^ zebrafish line and the IIMCB ZCF for assistance with the adult fish. This work was supported by an OPUS grant (no. 2020/37/B/NZ3/02345) from the National Science Centre, Poland. For the purpose of open access, the author has applied a CC-BY public copyright license to any author accepted manuscript (AAM) version arising from this submission.

## Author contributions

This protocol was developed by J.Z. The detailed procedure was written and edited by O.D., T.D., and J.Z. Resources and funding were secured by J.Z.

## Declaration of interests

The authors declare no competing interests.
