# Protocol for microinjection of rapamycin into the zebrafish habenula

**DOI:** 10.1016/j.xpro.2026.104407

**Published:** 2026-02-16

**Authors:** Olga Doszyn, Tomasz Dulski, Justyna Zmorzynska

## Main text

(STAR Protocols *6*, 103566; March 21, 2025)

Due to author oversight, in the originally published version of Figure 2A, the lateral view of the zebrafish habenulae schematic inadvertently suggested that the optic tectum area extends to cover the location of the diencephalon. The figure has been updated to distinguish the tegmentum and diencephalon areas. The authors regret the error and apologize for any confusion.

**Figure:**
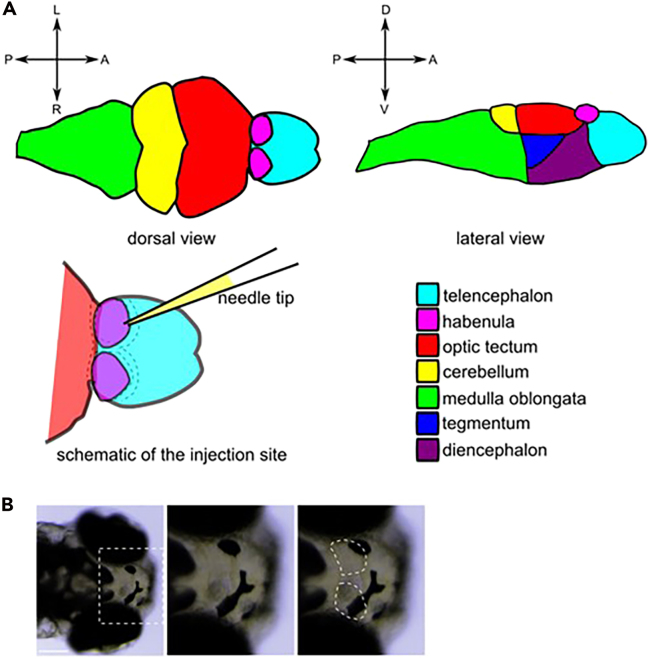
Figure 2. Habenula localization within the zebrafish brain (corrected)

**Figure:**
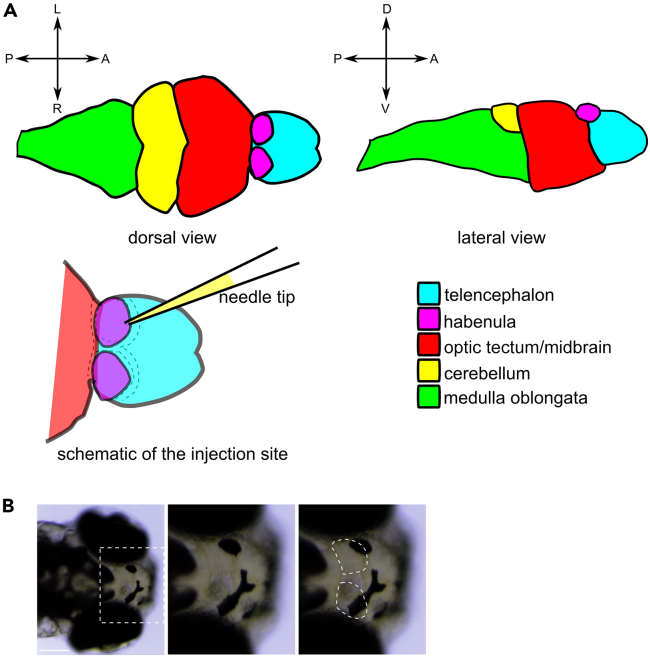
Figure 2. Habenula localization within the zebrafish brain (original)

